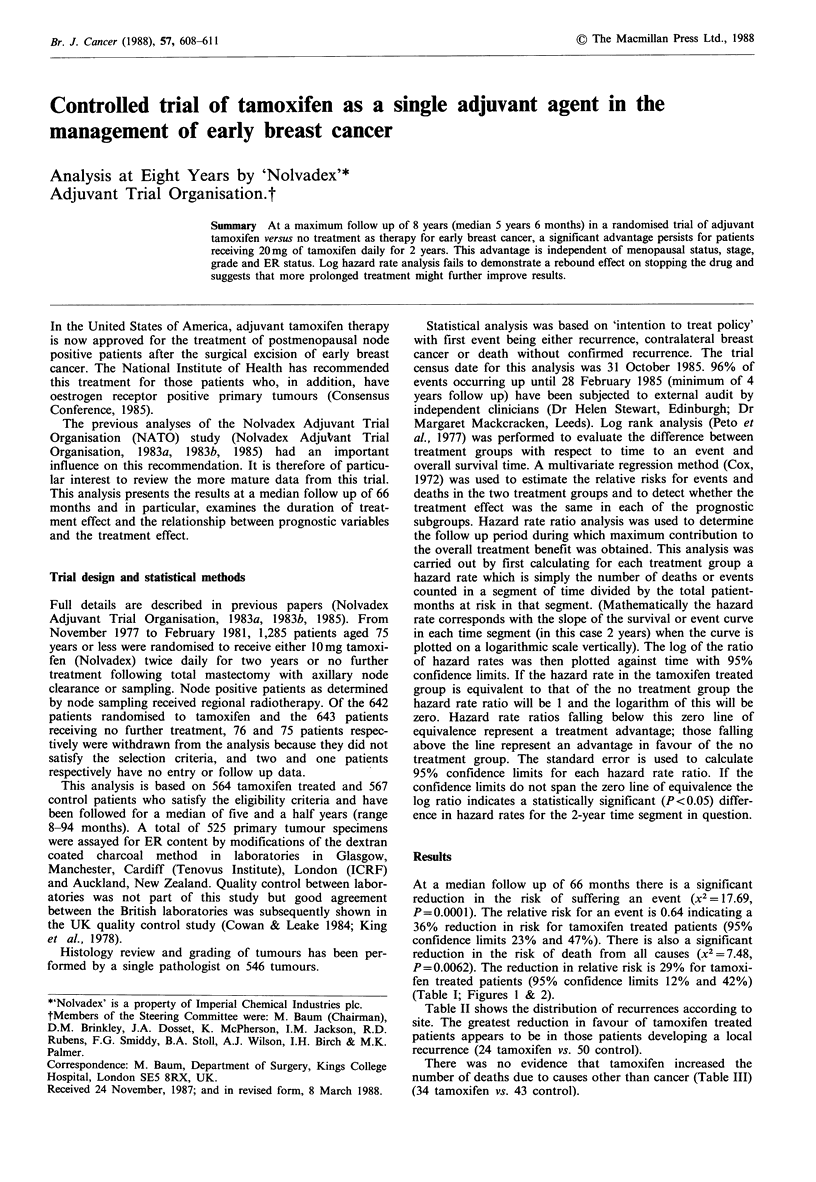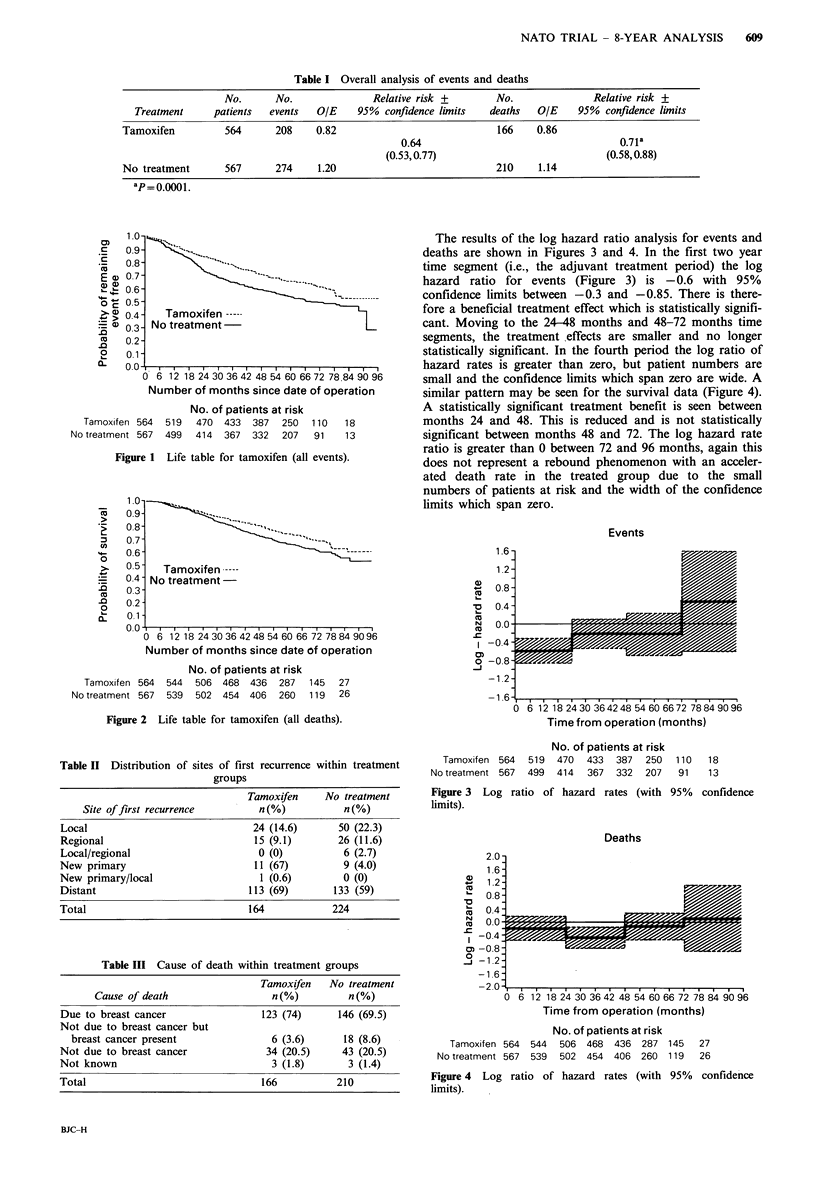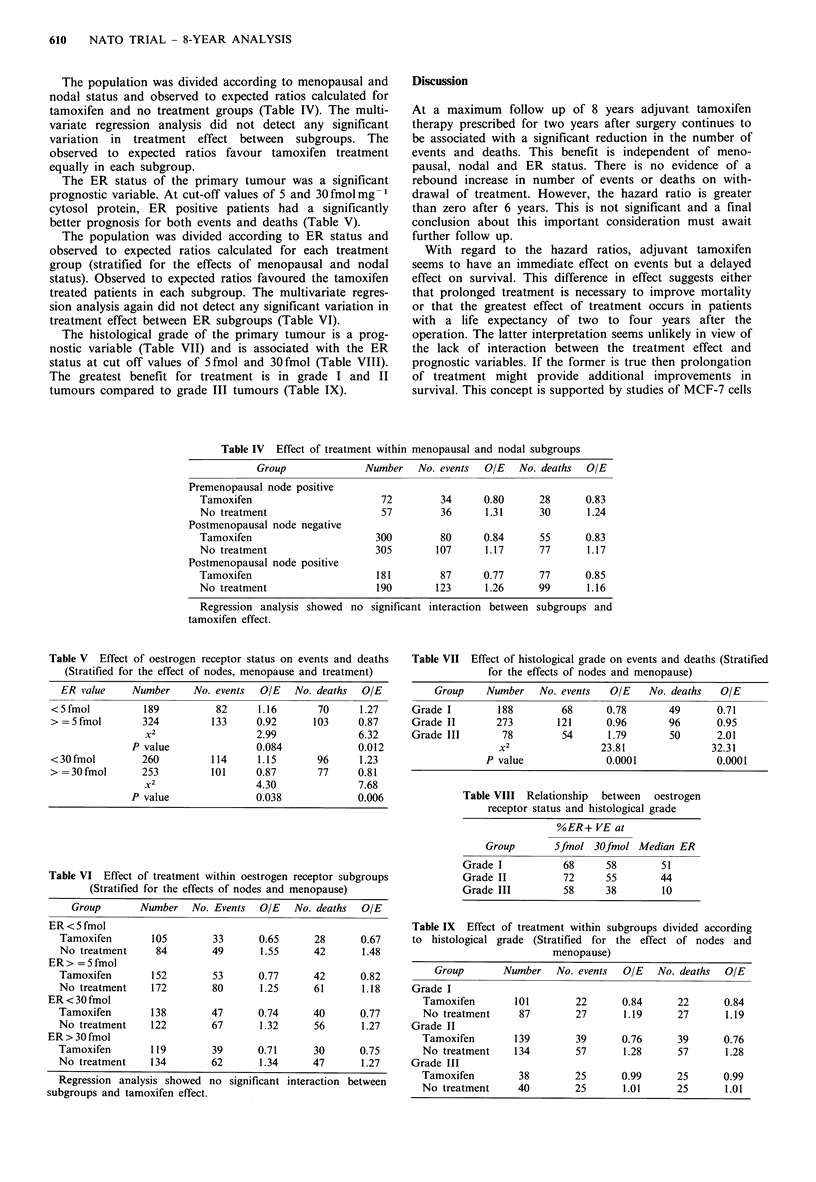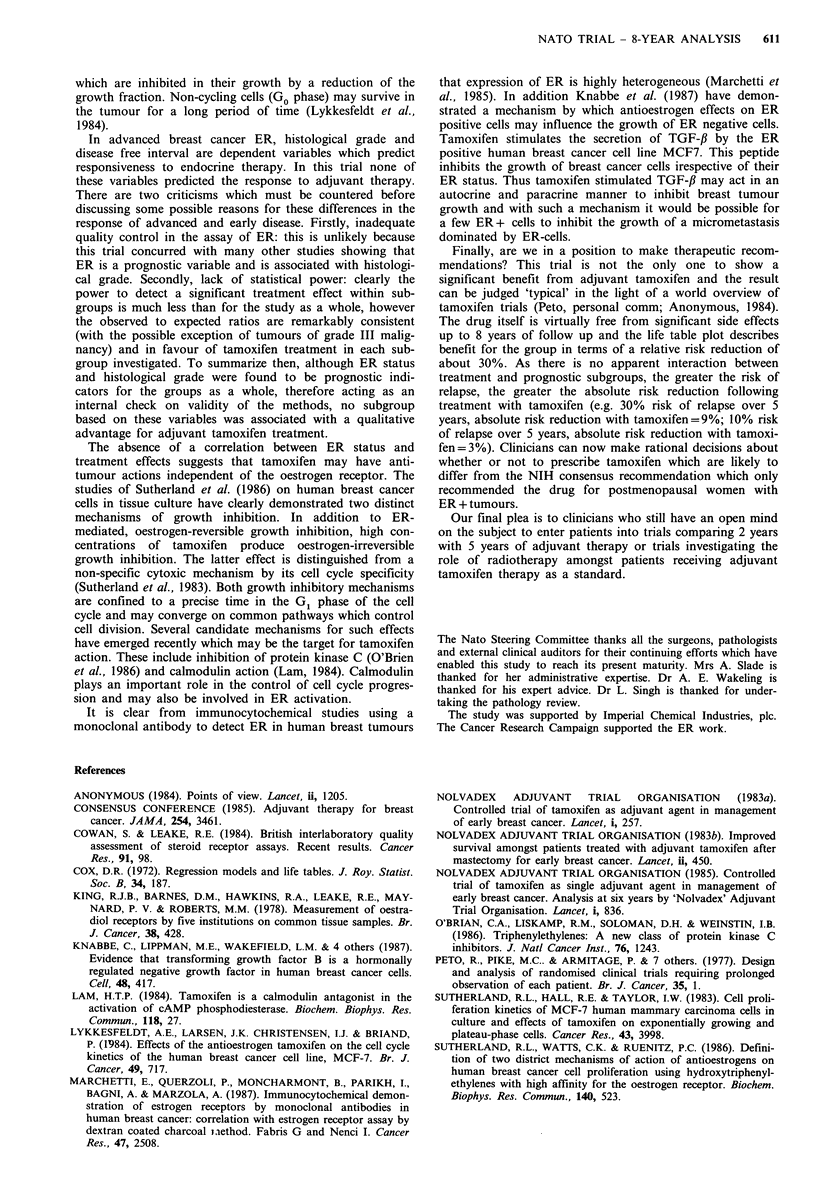# Controlled trial of tamoxifen as a single adjuvant agent in the management of early breast cancer. 'Nolvadex' Adjuvant Trial Organisation.

**DOI:** 10.1038/bjc.1988.138

**Published:** 1988-06

**Authors:** 

## Abstract

At a maximum follow up of 8 years (median 5 years 6 months) in a randomised trial of adjuvant tamoxifen versus no treatment as therapy for early breast cancer, a significant advantage persists for patients receiving 20 mg of tamoxifen daily for 2 years. This advantage is independent of menopausal status, stage, grade and ER status. Log hazard rate analysis fails to demonstrate a rebound effect on stopping the drug and suggests that more prolonged treatment might further improve results.


Br. J. Cancer (1988), 57, 608–611 © The Macmillan Press Ltd., 1988

### Controlled trial of tamoxifen as a single adjuvant agent in the management of early breast cancer

Analysis at Eight Years by 'Nolvadex'*
Adjuvant Trial Organisation.†

**Summary** At a maximum follow up of 8 years (median 5 years 6 months) in a randomised trial of adjuvant tamoxifen *versus* no treatment as therapy for early breast cancer, a significant advantage persists for patients receiving 20 mg of tamoxifen daily for 2 years. This advantage is independent of menopausal status, stage, grade and ER status. Log hazard rate analysis fails to demonstrate a rebound effect on stopping the drug and suggests that more prolonged treatment might further improve results.

In the United States of America, adjuvant tamoxifen therapy is now approved for the treatment of postmenopausal node positive patients after the surgical excision of early breast cancer. The National Institute of Health has recommended this treatment for those patients who, in addition, have oestrogen receptor positive primary tumours (Consensus Conference, 1985).

The previous analyses of the Nolvadex Adjuvant Trial Organisation (NATO) study (Nolvadex Adjuvant Trial Organisation, 1983*a*, 1983*b*, 1985) had an important influence on this recommendation. It is therefore of particular interest to review the more mature data from this trial. This analysis presents the results at a median follow up of 66 months and in particular, examines the duration of treatment effect and the relationship between prognostic variables and the treatment effect.

### Trial design and statistical methods

Full details are described in previous papers (Nolvadex Adjuvant Trial Organisation, 1983*a*, 1983*b*, 1985). From November 1977 to February 1981, 1,285 patients aged 75 years or less were randomised to receive either 10 mg tamoxifen (Nolvadex) twice daily for two years or no further treatment following total mastectomy with axillary node clearance or sampling. Node positive patients as determined by node sampling received regional radiotherapy. Of the 642 patients randomised to tamoxifen and the 643 patients receiving no further treatment, 76 and 75 patients respectively were withdrawn from the analysis because they did not satisfy the selection criteria, and two and one patients respectively have no entry or follow up data.

This analysis is based on 564 tamoxifen treated and 567 control patients who satisfy the eligibility criteria and have been followed for a median of five and a half years (range 8–94 months). A total of 525 primary tumour specimens were assayed for ER content by modifications of the dextran coated charcoal method in laboratories in Glasgow, Manchester, Cardiff (Tenovus Institute), London (ICRF) and Auckland, New Zealand. Quality control between laboratories was not part of this study but good agreement between the British laboratories was subsequently shown in the UK quality control study (Cowan & Leake 1984; King *et al.*, 1978).

Histology review and grading of tumours has been performed by a single pathologist on 546 tumours.

Statistical analysis was based on 'intention to treat policy' with first event being either recurrence, contralateral breast cancer or death without confirmed recurrence. The trial census date for this analysis was 31 October 1985. 96% of events occurring up until 28 February 1985 (minimum of 4 years follow up) have been subjected to external audit by independent clinicians (Dr Helen Stewart, Edinburgh; Dr Margaret Mackcracken, Leeds). Log rank analysis (Peto *et al.*, 1977) was performed to evaluate the difference between treatment groups with respect to time to an event and overall survival time. A multivariate regression method (Cox, 1972) was used to estimate the relative risks for events and deaths in the two treatment groups and to detect whether the treatment effect was the same in each of the prognostic subgroups. Hazard rate ratio analysis was used to determine the follow up period during which maximum contribution to the overall treatment benefit was obtained. This analysis was carried out by first calculating for each treatment group a hazard rate which is simply the number of deaths or events counted in a segment of time divided by the total patient-months at risk in that segment. (Mathematically the hazard rate corresponds with the slope of the survival or event curve in each time segment (in this case 2 years) when the curve is plotted on a logarithmic scale vertically). The log of the ratio of hazard rates was then plotted against time with 95% confidence limits. If the hazard rate in the tamoxifen treated group is equivalent to that of the no treatment group the hazard rate ratio will be 1 and the logarithm of this will be zero. Hazard rate ratios falling below this zero line of equivalence represent a treatment advantage; those falling above the line represent an advantage in favour of the no treatment group. The standard error is used to calculate 95% confidence limits for each hazard rate ratio. If the confidence limits do not span the zero line of equivalence the log ratio indicates a statistically significant ($P<0.05$) difference in hazard rates for the 2-year time segment in question.

### Results

At a median follow up of 66 months there is a significant reduction in the risk of suffering an event ($x^2=17.69$, $P=0.0001$). The relative risk for an event is 0.64 indicating a 36% reduction in risk for tamoxifen treated patients (95% confidence limits 23% and 47%). There is also a significant reduction in the risk of death from all causes ($x^2=7.48$, $P=0.0062$). The reduction in relative risk is 29% for tamoxifen treated patients (95% confidence limits 12% and 42%) (Table I; Figures 1 & 2).

Table II shows the distribution of recurrences according to site. The greatest reduction in favour of tamoxifen treated patients appears to be in those patients developing a local recurrence (24 tamoxifen *vs.* 50 control).

There was no evidence that tamoxifen increased the number of deaths due to causes other than cancer (Table III) (34 tamoxifen *vs.* 43 control).

*'Nolvadex' is a property of Imperial Chemical Industries plc.
†Members of the Steering Committee were: M. Baum (Chairman), D.M. Brinkley, J.A. Dosset, K. McPherson, I.M. Jackson, R.D. Rubens, F.G. Smiddy, B.A. Stoll, A.J. Wilson, I.H. Birch & M.K. Palmer.
Correspondence: M. Baum, Department of Surgery, Kings College Hospital, London SE5 8RX, UK.
Received 24 November, 1987; and in revised form, 8 March 1988.

**Table I** Overall analysis of events and deaths

| Treatment | No. patients | No. events | O/E | Relative risk ± 95% confidence limits | No. deaths | O/E | Relative risk ± 95% confidence limits |
|---|---|---|---|---|---|---|---|
| Tamoxifen | 564 | 208 | 0.82 | 0.64 (0.53, 0.77) | 166 | 0.86 | 0.71[a] (0.58, 0.88) |
| No treatment | 567 | 274 | 1.20 | | 210 | 1.14 | |

[a]$P = 0.0001$.

No. of patients at risk

| | | | | | | | | |
|---|---|---|---|---|---|---|---|---|
| Tamoxifen | 564 | 519 | 470 | 433 | 387 | 250 | 110 | 18 |
| No treatment | 567 | 499 | 414 | 367 | 332 | 207 | 91 | 13 |

**Figure 1** Life table for tamoxifen (all events).

No. of patients at risk

| | | | | | | | | |
|---|---|---|---|---|---|---|---|---|
| Tamoxifen | 564 | 544 | 506 | 468 | 436 | 287 | 145 | 27 |
| No treatment | 567 | 539 | 502 | 454 | 406 | 260 | 119 | 26 |

**Figure 2** Life table for tamoxifen (all deaths).

**Table II** Distribution of sites of first recurrence within treatment groups

| Site of first recurrence | Tamoxifen n(%) | No treatment n(%) |
|---|---|---|
| Local | 24 (14.6) | 50 (22.3) |
| Regional | 15 (9.1) | 26 (11.6) |
| Local/regional | 0 (0) | 6 (2.7) |
| New primary | 11 (67) | 9 (4.0) |
| New primary/local | 1 (0.6) | 0 (0) |
| Distant | 113 (69) | 133 (59) |
| Total | 164 | 224 |

**Table III** Cause of death within treatment groups

| Cause of death | Tamoxifen n(%) | No treatment n(%) |
|---|---|---|
| Due to breast cancer | 123 (74) | 146 (69.5) |
| Not due to breast cancer but breast cancer present | 6 (3.6) | 18 (8.6) |
| Not due to breast cancer | 34 (20.5) | 43 (20.5) |
| Not known | 3 (1.8) | 3 (1.4) |
| Total | 166 | 210 |

The results of the log hazard ratio analysis for events and deaths are shown in Figures 3 and 4. In the first two year time segment (i.e., the adjuvant treatment period) the log hazard ratio for events (Figure 3) is −0.6 with 95% confidence limits between −0.3 and −0.85. There is therefore a beneficial treatment effect which is statistically significant. Moving to the 24–48 months and 48–72 months time segments, the treatment effects are smaller and no longer statistically significant. In the fourth period the log ratio of hazard rates is greater than zero, but patient numbers are small and the confidence limits which span zero are wide. A similar pattern may be seen for the survival data (Figure 4). A statistically significant treatment benefit is seen between months 24 and 48. This is reduced and is not statistically significant between months 48 and 72. The log hazard rate ratio is greater than 0 between 72 and 96 months, again this does not represent a rebound phenomenon with an accelerated death rate in the treated group due to the small numbers of patients at risk and the width of the confidence limits which span zero.

No. of patients at risk

| | | | | | | | | |
|---|---|---|---|---|---|---|---|---|
| Tamoxifen | 564 | 519 | 470 | 433 | 387 | 250 | 110 | 18 |
| No treatment | 567 | 499 | 414 | 367 | 332 | 207 | 91 | 13 |

**Figure 3** Log ratio of hazard rates (with 95% confidence limits).

No. of patients at risk

| | | | | | | | | |
|---|---|---|---|---|---|---|---|---|
| Tamoxifen | 564 | 544 | 506 | 468 | 436 | 287 | 145 | 27 |
| No treatment | 567 | 539 | 502 | 454 | 406 | 260 | 119 | 26 |

**Figure 4** Log ratio of hazard rates (with 95% confidence limits).

The population was divided according to menopausal and nodal status and observed to expected ratios calculated for tamoxifen and no treatment groups (Table IV). The multivariate regression analysis did not detect any significant variation in treatment effect between subgroups. The observed to expected ratios favour tamoxifen treatment equally in each subgroup.

The ER status of the primary tumour was a significant prognostic variable. At cut-off values of 5 and 30 fmol $mg^{-1}$ cytosol protein, ER positive patients had a significantly better prognosis for both events and deaths (Table V).

The population was divided according to ER status and observed to expected ratios calculated for each treatment group (stratified for the effects of menopausal and nodal status). Observed to expected ratios favoured the tamoxifen treated patients in each subgroup. The multivariate regression analysis again did not detect any significant variation in treatment effect between ER subgroups (Table VI).

The histological grade of the primary tumour is a prognostic variable (Table VII) and is associated with the ER status at cut off values of 5 fmol and 30 fmol (Table VIII). The greatest benefit for treatment is in grade I and II tumours compared to grade III tumours (Table IX).

### Discussion

At a maximum follow up of 8 years adjuvant tamoxifen therapy prescribed for two years after surgery continues to be associated with a significant reduction in the number of events and deaths. This benefit is independent of menopausal, nodal and ER status. There is no evidence of a rebound increase in number of events or deaths on withdrawal of treatment. However, the hazard ratio is greater than zero after 6 years. This is not significant and a final conclusion about this important consideration must await further follow up.

With regard to the hazard ratios, adjuvant tamoxifen seems to have an immediate effect on events but a delayed effect on survival. This difference in effect suggests either that prolonged treatment is necessary to improve mortality or that the greatest effect of treatment occurs in patients with a life expectancy of two to four years after the operation. The latter interpretation seems unlikely in view of the lack of interaction between the treatment effect and prognostic variables. If the former is true then prolongation of treatment might provide additional improvements in survival. This concept is supported by studies of MCF-7 cells

**Table IV** Effect of treatment within menopausal and nodal subgroups

| *Group* | *Number* | *No. events* | *O/E* | *No. deaths* | *O/E* |
|---|---|---|---|---|---|
| Premenopausal node positive | | | | | |
| Tamoxifen | 72 | 34 | 0.80 | 28 | 0.83 |
| No treatment | 57 | 36 | 1.31 | 30 | 1.24 |
| Postmenopausal node negative | | | | | |
| Tamoxifen | 300 | 80 | 0.84 | 55 | 0.83 |
| No treatment | 305 | 107 | 1.17 | 77 | 1.17 |
| Postmenopausal node positive | | | | | |
| Tamoxifen | 181 | 87 | 0.77 | 77 | 0.85 |
| No treatment | 190 | 123 | 1.26 | 99 | 1.16 |

Regression analysis showed no significant interaction between subgroups and tamoxifen effect.

**Table V** Effect of oestrogen receptor status on events and deaths (Stratified for the effect of nodes, menopause and treatment)

| *ER value* | *Number* | *No. events* | *O/E* | *No. deaths* | *O/E* |
|---|---|---|---|---|---|
| <5 fmol | 189 | 82 | 1.16 | 70 | 1.27 |
| > = 5 fmol | 324 | 133 | 0.92 | 103 | 0.87 |
| | $x^2$ | | 2.99 | | 6.32 |
| | *P* value | | 0.084 | | 0.012 |
| <30 fmol | 260 | 114 | 1.15 | 96 | 1.23 |
| > = 30 fmol | 253 | 101 | 0.87 | 77 | 0.81 |
| | $x^2$ | | 4.30 | | 7.68 |
| | *P* value | | 0.038 | | 0.006 |

**Table VI** Effect of treatment within oestrogen receptor subgroups (Stratified for the effects of nodes and menopause)

| *Group* | *Number* | *No. Events* | *O/E* | *No. deaths* | *O/E* |
|---|---|---|---|---|---|
| ER < 5 fmol | | | | | |
| Tamoxifen | 105 | 33 | 0.65 | 28 | 0.67 |
| No treatment | 84 | 49 | 1.55 | 42 | 1.48 |
| ER > = 5 fmol | | | | | |
| Tamoxifen | 152 | 53 | 0.77 | 42 | 0.82 |
| No treatment | 172 | 80 | 1.25 | 61 | 1.18 |
| ER < 30 fmol | | | | | |
| Tamoxifen | 138 | 47 | 0.74 | 40 | 0.77 |
| No treatment | 122 | 67 | 1.32 | 56 | 1.27 |
| ER > 30 fmol | | | | | |
| Tamoxifen | 119 | 39 | 0.71 | 30 | 0.75 |
| No treatment | 134 | 62 | 1.34 | 47 | 1.27 |

Regression analysis showed no significant interaction between subgroups and tamoxifen effect.

**Table VII** Effect of histological grade on events and deaths (Stratified for the effects of nodes and menopause)

| *Group* | *Number* | *No. events* | *O/E* | *No. deaths* | *O/E* |
|---|---|---|---|---|---|
| Grade I | 188 | 68 | 0.78 | 49 | 0.71 |
| Grade II | 273 | 121 | 0.96 | 96 | 0.95 |
| Grade III | 78 | 54 | 1.79 | 50 | 2.01 |
| | $x^2$ | | 23.81 | | 32.31 |
| | *P* value | | 0.0001 | | 0.0001 |

**Table VIII** Relationship between oestrogen receptor status and histological grade

| | *%ER+VE at* | | |
|---|---|---|---|
| *Group* | *5 fmol* | *30 fmol* | *Median ER* |
| Grade I | 68 | 58 | 51 |
| Grade II | 72 | 55 | 44 |
| Grade III | 58 | 38 | 10 |

**Table IX** Effect of treatment within subgroups divided according to histological grade (Stratified for the effect of nodes and menopause)

| *Group* | *Number* | *No. events* | *O/E* | *No. deaths* | *O/E* |
|---|---|---|---|---|---|
| Grade I | | | | | |
| Tamoxifen | 101 | 22 | 0.84 | 22 | 0.84 |
| No treatment | 87 | 27 | 1.19 | 27 | 1.19 |
| Grade II | | | | | |
| Tamoxifen | 139 | 39 | 0.76 | 39 | 0.76 |
| No treatment | 134 | 57 | 1.28 | 57 | 1.28 |
| Grade III | | | | | |
| Tamoxifen | 38 | 25 | 0.99 | 25 | 0.99 |
| No treatment | 40 | 25 | 1.01 | 25 | 1.01 |

which are inhibited in their growth by a reduction of the growth fraction. Non-cycling cells ($G_0$ phase) may survive in the tumour for a long period of time (Lykkesfeldt *et al.*, 1984).

In advanced breast cancer ER, histological grade and disease free interval are dependent variables which predict responsiveness to endocrine therapy. In this trial none of these variables predicted the response to adjuvant therapy. There are two criticisms which must be countered before discussing some possible reasons for these differences in the response of advanced and early disease. Firstly, inadequate quality control in the assay of ER: this is unlikely because this trial concurred with many other studies showing that ER is a prognostic variable and is associated with histological grade. Secondly, lack of statistical power: clearly the power to detect a significant treatment effect within subgroups is much less than for the study as a whole, however the observed to expected ratios are remarkably consistent (with the possible exception of tumours of grade III malignancy) and in favour of tamoxifen treatment in each subgroup investigated. To summarize then, although ER status and histological grade were found to be prognostic indicators for the groups as a whole, therefore acting as an internal check on validity of the methods, no subgroup based on these variables was associated with a qualitative advantage for adjuvant tamoxifen treatment.

The absence of a correlation between ER status and treatment effects suggests that tamoxifen may have antitumour actions independent of the oestrogen receptor. The studies of Sutherland *et al.* (1986) on human breast cancer cells in tissue culture have clearly demonstrated two distinct mechanisms of growth inhibition. In addition to ER-mediated, oestrogen-reversible growth inhibition, high concentrations of tamoxifen produce oestrogen-irreversible growth inhibition. The latter effect is distinguished from a non-specific cytoxic mechanism by its cell cycle specificity (Sutherland *et al.*, 1983). Both growth inhibitory mechanisms are confined to a precise time in the $G_1$ phase of the cell cycle and may converge on common pathways which control cell division. Several candidate mechanisms for such effects have emerged recently which may be the target for tamoxifen action. These include inhibition of protein kinase C (O'Brien *et al.*, 1986) and calmodulin action (Lam, 1984). Calmodulin plays an important role in the control of cell cycle progression and may also be involved in ER activation.

It is clear from immunocytochemical studies using a monoclonal antibody to detect ER in human breast tumours that expression of ER is highly heterogeneous (Marchetti *et al.*, 1985). In addition Knabbe *et al.* (1987) have demonstrated a mechanism by which antioestrogen effects on ER positive cells may influence the growth of ER negative cells. Tamoxifen stimulates the secretion of TGF-$\beta$ by the ER positive human breast cancer cell line MCF7. This peptide inhibits the growth of breast cancer cells irespective of their ER status. Thus tamoxifen stimulated TGF-$\beta$ may act in an autocrine and paracrine manner to inhibit breast tumour growth and with such a mechanism it would be possible for a few ER+ cells to inhibit the growth of a micrometastasis dominated by ER-cells.

Finally, are we in a position to make therapeutic recommendations? This trial is not the only one to show a significant benefit from adjuvant tamoxifen and the result can be judged 'typical' in the light of a world overview of tamoxifen trials (Peto, personal comm; Anonymous, 1984). The drug itself is virtually free from significant side effects up to 8 years of follow up and the life table plot describes benefit for the group in terms of a relative risk reduction of about 30%. As there is no apparent interaction between treatment and prognostic subgroups, the greater the risk of relapse, the greater the absolute risk reduction following treatment with tamoxifen (e.g. 30% risk of relapse over 5 years, absolute risk reduction with tamoxifen = 9%; 10% risk of relapse over 5 years, absolute risk reduction with tamoxifen = 3%). Clinicians can now make rational decisions about whether or not to prescribe tamoxifen which are likely to differ from the NIH consensus recommendation which only recommended the drug for postmenopausal women with ER + tumours.

Our final plea is to clinicians who still have an open mind on the subject to enter patients into trials comparing 2 years with 5 years of adjuvant therapy or trials investigating the role of radiotherapy amongst patients receiving adjuvant tamoxifen therapy as a standard.

The Nato Steering Committee thanks all the surgeons, pathologists and external clinical auditors for their continuing efforts which have enabled this study to reach its present maturity. Mrs A. Slade is thanked for her administrative expertise. Dr A. E. Wakeling is thanked for his expert advice. Dr L. Singh is thanked for undertaking the pathology review.

The study was supported by Imperial Chemical Industries, plc. The Cancer Research Campaign supported the ER work.

### References

ANONYMOUS (1984). Points of view. *Lancet*, **ii**, 1205.

CONSENSUS CONFERENCE (1985). Adjuvant therapy for breast cancer. *JAMA*, **254**, 3461.

COWAN, S. & LEAKE, R.E. (1984). British interlaboratory quality assessment of steroid receptor assays. Recent results. *Cancer Res.*, **91**, 98.

COX, D.R. (1972). Regression models and life tables. *J. Roy. Statist. Soc. B*, **34**, 187.

KING, R.J.B., BARNES, D.M., HAWKINS, R.A., LEAKE, R.E., MAYNARD, P. V. & ROBERTS, M.M. (1978). Measurement of oestradiol receptors by five institutions on common tissue samples. *Br. J. Cancer*, **38**, 428.

KNABBE, C., LIPPMAN, M.E., WAKEFIELD, L.M. & 4 others (1987). Evidence that transforming growth factor B is a hormonally regulated negative growth factor in human breast cancer cells. *Cell*, **48**, 417.

LAM, H.T.P. (1984). Tamoxifen is a calmodulin antagonist in the activation of cAMP phosphodiesterase. *Biochem. Biophys. Res. Commun.*, **118**, 27.

LYKKESFELDT, A.E., LARSEN, J.K. CHRISTENSEN, I.J. & BRIAND, P. (1984). Effects of the antioestrogen tamoxifen on the cell cycle kinetics of the human breast cancer cell line, MCF-7. *Br. J. Cancer*, **49**, 717.

MARCHETTI, E., QUERZOLI, P., MONCHARMONT, B., PARIKH, I., BAGNI, A. & MARZOLA, A. (1987). Immunocytochemical demonstration of estrogen receptors by monoclonal antibodies in human breast cancer: correlation with estrogen receptor assay by dextran coated charcoal method. Fabris G and Nenci I. *Cancer Res.*, **47**, 2508.

NOLVADEX ADJUVANT TRIAL ORGANISATION (1983*a*). Controlled trial of tamoxifen as adjuvant agent in management of early breast cancer. *Lancet*, **i**, 257.

NOLVADEX ADJUVANT TRIAL ORGANISATION (1983*b*). Improved survival amongst patients treated with adjuvant tamoxifen after mastectomy for early breast cancer. *Lancet*, **ii**, 450.

NOLVADEX ADJUVANT TRIAL ORGANISATION (1985). Controlled trial of tamoxifen as single adjuvant agent in management of early breast cancer. Analysis at six years by 'Nolvadex' Adjuvant Trial Organisation. *Lancet*, **i**, 836.

O'BRIAN, C.A., LISKAMP, R.M., SOLOMAN, D.H. & WEINSTIN, I.B. (1986). Triphenylethylenes: A new class of protein kinase C inhibitors. *J. Natl Cancer Inst.*, **76**, 1243.

PETO, R., PIKE, M.C.. & ARMITAGE, P. & 7 others. (1977). Design and analysis of randomised clinical trials requiring prolonged observation of each patient. *Br. J. Cancer*, **35**, 1.

SUTHERLAND, R.L., HALL, R.E. & TAYLOR, I.W. (1983). Cell proliferation kinetics of MCF-7 human mammary carcinoma cells in culture and effects of tamoxifen on exponentially growing and plateau-phase cells. *Cancer Res.*, **43**, 3998.

SUTHERLAND, R.L., WATTS, C.K. & RUENITZ, P.C. (1986). Definition of two district mechanisms of action of antioestrogens on human breast cancer cell proliferation using hydroxytriphenylethylenes with high affinity for the oestrogen receptor. *Biochem. Biophys. Res. Commun.*, **140**, 523.